# Limits of Tumor Detectability in Nuclear Medicine and PET

**DOI:** 10.4274/Mirt.138

**Published:** 2012-04-01

**Authors:** Yusuf Emre Erdi

**Affiliations:** 1 Memorial Sloan Kettering Cancer Center, Department of Medical Physics, New York, USA

**Keywords:** nuclear medicine, PET, lesion, detection, limits

## Abstract

**Objective:** Nuclear medicine is becoming increasingly important in the early detection of malignancy. The advantage of nuclear medicine over other imaging modalities is the high sensitivity of the gamma camera. Nuclear medicine counting equipment has the capability of detecting levels of radioactivity which exceed background levels by as little as 2.4 to 1. This translates to only a few hundred counts per minute on a regular gamma camera or as few as 3 counts per minute when using coincidence detection on a positron emission tomography (PET) camera.

**Material and Methods:** We have experimentally measured the limits of detectability using a set of hollow spheres in a Jaszczak phantom at various tumor-to-background ratios. Imaging modalities for this work were (1) planar, (2) SPECT, (3) PET, and (4) planar camera with coincidence detection capability (MCD).

**Results:** When there is no background (infinite contrast) activity present, the detectability of tumors is similar for PET and planar imaging. With the presence of the background activity , PET can detect objects in an order of magnitude smaller in size than that can be seen by conventional planar imaging especially in the typical clinical low (3:1) T/B ratios. The detection capability of the MCD camera lies between a conventional nuclear medicine (planar / SPECT) scans and the detection capability of a dedicated PET scanner.

**Conclusion: ** Among nuclear medicine’s armamentarium, PET is the closest modality to CT or MR imaging in terms of limits of detection. Modern clinical PET scanners have a resolution limit of 4 mm, corresponding to the detection of tumors with a volume of 0.2 ml (7 mm diameter) in 5:1 T/B ratio. It is also possible to obtain better resolution limits with dedicated brain and animal scanners. The future holds promise in development of new detector materials, improved camera design, and new reconstruction algorithms which will improve sensitivity, resolution, contrast, and thereby further diminish the limits of tumor detectability.

**Conflict of interest:**None declared.

## INTRODUCTION

Even though with recent advances in computer technology and its use in the medical community; most medical images are still being evaluated by a human observer. If the image is of high contrast, the observer will experience little difficulty to distinguish tumors from their background, probably accurately estimating their size and shape. Often, however, the image will be of low contrast, blurred by noise. In low contrast images the observer works at the limit of detection capacity. 

Nuclear medicine is becoming increasingly important in the early detection of malignancy. The advantage of nuclear medicine over other imaging modalities is the high sensitivity of the gamma camera. Nuclear medicine counting equipment has the capability of detecting levels of radioactivity which exceed background levels by at least 2.4 to 1 fold. This means few hundred counts per minute on a regular gamma camera or as few as 3 counts per minute when using coincidence detection on a positron emission tomography (PET) camera. 

In nuclear medicine imaging, the difference in signal between tumor and the surrounding normal tissue depends upon the tumor-localizing agent. Tumor-to-background (T/B) ratios are found to be anything from 1:1 to > 10:1 with radiolabeled antibodies (1), radioiodine in thyroid cancer (2), dependent upon the time of imaging post administration. It is also possible to obtain improved sensitivity using site-specific agents, such as, somatostatin receptors (3). In spite of the superior sensitivity and T/B ratios implicit in nuclear medicine studies, relative to alternative imaging modalities such as computed tomography (CT), magnetic resonance (MR), ultrasound and mammography, radiologists frequently express their dissatisfaction with tumor imaging using gamma cameras. There are two causes of this dissatisfaction. 

(1) The resolution of gamma camera images is lower than achievable by CT or MRI. The intrinsic resolution of a modern sodium iodide gamma camera, expressed as the full width at half maximum (FWHM) for a line source of infinitesimal thickness, is approximately 3.5 mm. But the true resolution of a clinical study is degraded by the addition of a collimator, distance from the detector, photon scatter and patient motion. For example, the FWHM for Tc-99m degrades from 7.5 mm to 19.1 mm where the depth of the source in water changes from 2 cm to 22 cm (4). The lower resolution of gamma camera images is the cause of their blurry appearance.

(2) Nuclear medicine images are functional images, differentiating between tumors and/or organs on the basis of specific binding or internalization versus non-specific association and blood pool activity. As such, the anatomical information present in gamma camera images is very poor compared to CT or MR, and highly dependent upon the imaging agent. 

In spite of these disadvantages of nuclear medicine imaging, it is possible to pick up tumors not seen by other modalities, because of the ability to differentiate tissues on the basis of function. For example, F-18-fluoro-2-deoxy-D-glucose (FDG-18) PET imaging has the ability to distinguish between malignant, benign and edematous tissue, not readily detectable on CT scans (5). Furthermore, the poorer intrinsic resolution of gamma cameras does not result in lower limits of tumor detectability of nuclear medicine techniques relative to other imaging modalities. Tumors can be detected, well below the spatial limit of resolution of the gamma camera, provided the T/B ratio is adequately high. Even a tumor of <1 mm diameter is detectable, provided both the background activity is negligible and the number of radioactive atoms associated with the tumor sufficient to exceed by more than 2.5 times the background count rate. Objects smaller in sizes than twice the FWHM of a gamma camera are rendered in the image larger than actual size (6). This effect is referred to as the partial volume effect, and results in a well-known underestimate of the specific activity associated with a tumor.

An extremely important diagnostic question in nuclear medicine is the determination of the limits of detectability of tumors under clinical conditions. This will depend upon numerous parameters:

(1) the tumor to background ratio (7,8)

(2) the imaging isotope (9,10,11)

(3) the depth and location of the tumor (12)

(4) the total number of counts in the image (8)

(5) the properties of gamma camera imaging system, e.g. collimator (13)

(6) the image processing e.g. reconstruction algorithm, filters, cut-off etc.(14)

Extensive theoretical analysis of the factors affecting tumor detectability have been performed by (7,8,13).

Rockoff et al. (7) developed a method to analyze limits of detection for tumors in planar camera imaging. Assuming a Gaussian-shaped tumor signal, they calculated signal-to-noise (SNR) ratio of a tumor as:

SNR = | U-1 |. μ. √CB.V.e-μd / (1-e-μd).√A_eff_ Eq. 1 

where

U: uptake (tumor-to-background) 

ratio

V: tumor volume 

d: depth

C_B_: Count density in the background

A_eff_: Effective area of the tumor mass as presented in the gamma camera image 

**Figure 1:**
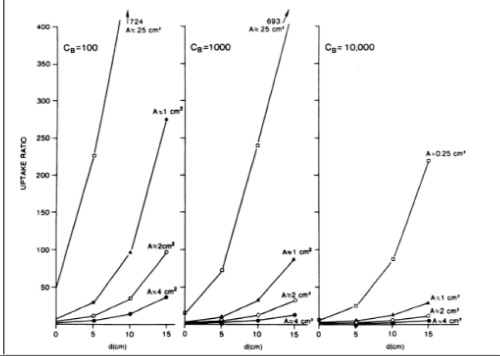
Computed T/B ratios needed for imaging of various sizetumors in a 30-cm-thick patient. Count densities (CB) of 100, 1.000and 10.000 at depths (d) ranging from the surface (0 cm) to15 cm. A is the area of tumor. (Reprinted from Rockoff et al. (7) bypermission from American Association for Cancer Research

μ : Linear attenuation coefficientSolving Eq. 1 for U using various parameters yields the family of curves given in Figure 1. It is apparent from Eq.1 that, for small tumors (A= 0.25 or 1.0 cm2), deep tumors (d ≥5 cm), and/or low count densities (C_B_ ≤1000), there is a challenging requirement on the uptake ratio, tending to be over 5 in order to be able to detect the tumor.

Goodenough and Atkins (13) have extended the work of Rockoff et al. (7) by performing simulation studies with In-111. In that study, they investigated the relation among collimator resolution, intrinsic resolution, and T/B ratio. They also searched noise characteristics of single photon emission computed tomography (SPECT) and planar images and concluded that if the planar image was smoothed to yield the same resolution as in the SPECT image with a slice thickness equal to the spatial resolution, then the noise variances of the two data sets were comparable. 

Bradwell et al. (8) investigated the factors which affect tumor detectability using planar gamma camera imaging of an In-111 radiolabeled antibody in clinical settings. They adapted the radioimmunolocalization model developed by Rockoff et al. (7) to determine the practical limitations in relation to the present state of art. Among the 11 parameters of their radioimmunolocalization model, they concluded the most sensitive parameter affecting detectability was the T/B ratio. The second most important parameter was the total number of counts detected from the tumor. This group presented useful graphs which show the smallest detectable tumor using In-111 at various T/B ratios and count rates.

## MATERIALS AND METHODS

We have experimentally measured the limits of detectability using a set of hollow spheres in a Jaszczak phantom at various T/B ratios. Imaging modalities for this work were (1) planar imaging with conventional gamma camera, (2) SPECT, (3) PET, and (4) planar imaging with a camera which has coincidence detection capability. General characteristics of these imaging modalities will be presented below briefly.

(1) Planar camera provides a high count image of the patient from a single angle, e.g., a typical count rate of 178 cpm/μCi of 99mTc with the low energy high resolution (LEHR) collimator. In-air resolution typically is about 8.5 mm at 20 cm with Genesys (ADAC, Milpitas CA) camera. 

(2) The resolution of SPECT is 10% lower than that of planar imaging, due to the distance between patient and detector, imperfections in the center of rotation correction, and reconstruction artifacts. 

(3) The lack of collimation on a PET scanner results in a system sensitivity which is approximately 10-100 fold greater than SPECT camera. The resolution is also higher with a FWHM of 4.2 mm for 18F on central axis of the GE Advance camera in septa-in (2D) mode. This resolution, however, is slightly lower (4.6 mm) when the camera is used in septa-out (3D) mode (15).

(4) Gamma cameras with coincidence detection function as a standard planar or SPECT camera with the added coincidence circuitry. We have used an EPIC-Vertex (ADAC, Milpitas CA) camera with MCD (Molecular Coincidence Detection) option. The system has a count-rate limit which is about 2.2 M/sec which makes impossible accurately imaging activities more than 2 mCi in the field-of-view. The spatial resolution of this system is 6.4 mm which approaches that of dedicated PET scanners. In coincidence detection mode, there is no collimator attached to the system, so that, the detection of spatially sensitive scattered photons may be increased.

Spheres (tumors) of different sizes were imaged with Tc-99m, I-131, and F-18 at T/B ratios of 10:1, 5:1, 3:1 and no background (NoB). A Jaszczak phantom (Data Spectrum, NC) was used, containing spheres ranging in size from 0.2 to 12 cc. All spheres were filled with the same specific activity of either Tc-99m, I-131, or F-18, corresponding to levels of activity typical in clinical studies. In each study, the spheres were first imaged in the cylindrical Jaszczak filled with water, but with NoB activity present. Then, each study was repeated after progressively adding activity into phantom background. 

Planar and SPECT images were acquired on a dual headed Genesys (ADAC, Milpitas CA) camera. For the planar study, the camera was placed in the anterior - posterior configuration. Only the anterior head was used in the analysis. The extrinsic flood uniformity, measured with a Co-57 flood source, was < 3.5%. A LEHR and high energy general purpose (HEGP) collimator was used for the Tc-99m and I-131 work respectively. The Jaszczak phantom was positioned on the couch in the upright position, i.e., with the axis of the cylinder parallel to the collimator holes. The centers of all spheres were at 5 cm depth. Planar images of the I-131 were acquired for 10 minutes. 

PET data were acquired for 10 minutes on both Advance (GEMS, Milwaukee, WI) and Vertex-MCD (ADAC, Milpitas CA) imaging systems. There was approximately 3 μCi/ml of F-18 in the spheres. Both data sets were reconstructed using a Hann filter. Data from Advance scanner were attenuation corrected using measured transmission data, while MCD data were corrected using the Chang analytic method. Since the purpose of this investigation was to simulate clinical conditions, experimental data had not been optimized for filter selection or reconstruction parameters.

## RESULTS

**Figure 2:**
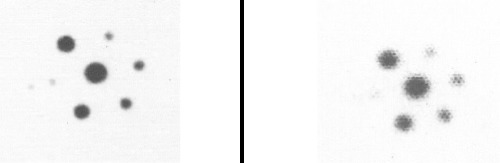
Spheres with Tc-99m (left) and I-131 (right) without abackground activity. Largest sphere is 11.9 ml and smallest is 0.2 ml

All hot spheres can be visualized when no background activity is present. However, the presence of larger hot objects in a phantom, can obscure visualization of very small objects, as for example observed (Figure 2) with the 0.2 cc sphere in our I-131 SPECT study (Table 1). This is a consequence of backprojection artifacts and therefore can be slightly lessened by the choice of reconstruction filter and cutoff. Once activity is added into the background, the tumor contrast is reduced (Table 2). At a 10:1, tumor to background ratio, the smallest discernible sphere becomes 1.5 cc., at 5:1 spheres < 5.8 cc became invisible by both planar and SPECT imaging for I-131 (Table 1). Note that SPECT, due to its noisier and more mottled appearance, offers no advantages over planar imaging unless studying overlapping structures (16,17) which may have an implication of overestimation of activity for a small tumor with overlaying tissues. Although the images of Tc-99m appear more visually pleasing (due to less septal penetration) than I-131, the limits of detectability for Tc-99m are similar to I-131 as shown in Tables 1 and 3. Tc-99m does offer an advantage (Table 3) in contrast and detection at 5:1 T/B ratio which is the most relevant clinically.

The measured image contrast defined as the maximum number of counts within each lesion region-of-interest (ROI) divided by the average number of counts in the background is given in Table 2 for Tc-99m planar, I-131 planar and I-131 SPECT images. The measured contrast is much lower than actual tumor-to-background ratio and becomes smaller with decreasing sphere size. This is a consequence of 3 factors:

1) Planar images are projections of detected events through the entire phantom and therefore consist of an average counts emanating from the tumor and a progressively larger background with diminishing sphere size.

2) The higher tumor activity results in a greater probability of photon cross-talk into the neighboring regions due to scatter. This probability of out-scatter increases with decreasing tumor diameter.

3) Due to the greater partial volume effect in the smaller tumor, fewer counts are detected and poorer count statistics are obtained per unit specific activity. 

For SPECT imaging, #1 above dose not apply, except that backprojection reconstruction smears (smooths) the activity through the tumor cross-section and leads to loss in image contrast along with choice of filter, cut-off frequencies, and attenuation correction.

PET improves the tumor detectability by almost an order of magnitude (Table 4), as a consequence of the sharp reduction in the FWHM. It can accurately reproduce the contrast of the object where the object is larger than 2 times FWHM of the system. Similar results are given for the MCD with a different set of spheres (Table 5).

**Summary of the Results**

When there is no background (infinite contrast) activity present, the detectability of tumors is similar for PET and planar imaging. With the presence of the background activity, PET can detect objects an order of magnitude smaller in size than can be seen by conventional planar imaging, especially in the typical clinical low (3:1) T/B ratios. The detection capability of the MCD camera lies between a conventional nuclear medicine (planar/SPECT) scans and the detection capability of a dedicated PET scanner. Table 6 summarizes the detection capabilities of different modalities in the presence of a high-background activity.

## DISCUSSION

The limits of detectability of tumors under clinical conditions may depend on many parameters, e.g., location of the tumor and imaging isotope. Clinical investigations in nuclear medicine demonstrates that minimum lesion detectability is about 1.5 cm in diameter (12,18,19,20). Assuming a spherical shape, this corresponds 1.77 ml of lesion volume. This agrees well with our experimental results, since we showed that boundary of detection is between 1.5 ml and 2.0 ml for Tc-99m planar imaging at 5:1 T/B ratio. When there is an exceptionally high T/B, e.g, (tumor/serum = 13.6) ratio obtained in clinical studies with I-131 labeled G250 antibody for renal cell carcinoma, limits of detection improves 2-fold to be at 8 mm for a minimum detectable tumor (21). This tumor diameter corresponds to a volume of 0.26 ml and agrees with the experimental data since, for I-131 planar imaging, a tumor volume of 0.2 ml should be visible on the images(Table 1). For I-131 SPECT imaging, it has been reported that a minimum detectable tumor diameter is about 1.0 cm, which is 0.53 ml for very high T/B ratios (tumor/serum =23:1) (22). At this level, the effect of background activity is negligible and Table 1 depicts that tumors of this size should be clearly visible, supporting the finding of this clinical investigation by Welt et al. (22).

The basis for the discrimination of malignant tissue in other modalities is different than in nuclear medicine. For example, CT uses the differential attenuation of x-rays through tissue. The limit of detectability in CT is a difference in attenuation of about 5 Hounsfield units, which corresponds to a difference of 5/1000th in x-ray attenuation of one ray through the body, relative to the adjacent. The reason for the high quality of CT images, in spite of the intrinsic low contrast between tissue structures, is the use of high x-ray fluence. The typical dose delivered using a typical chest CT with and without contrast is 4 rem (23) corresponding to a fluence of 1011 photons/cm2. This number of photons facilitates to separate small differences in tissue attenuation with 1 mm resolution. Due to the physical limitations, however, the minimum lesion size that can be measured with CT is about 3 mm (24). Modern MR imaging systems demonstrate similar lesion detection limits (25). 

Among nuclear medicine’s armamentarium, PET is the closest modality to CT or MR imaging in terms of size limits of detection. Modern clinical PET scanners have a resolution limit of 4 mm, corresponding to the detection of tumors with a volume of 0.2 ml (7 mm diameter) in 5:1 T/B ratio (Table 4). It is also possible to obtain better resolution limits with dedicated brain and animal scanners. The future holds promise in development of new detector materials, improved camera design, and new reconstruction algorithms which will improve sensitivity, resolution, contrast, and thereby further diminish the limits of tumor detectability.

## Figures and Tables

**Table 1:**
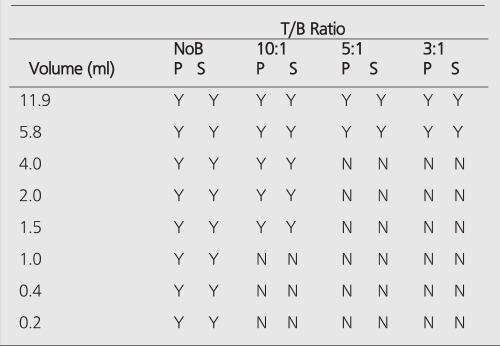
Limits of detection for I-131. Y denotes that sphere isvisible on planar (P) and SPECT (S) images, N means otherwise

**Table 2:**
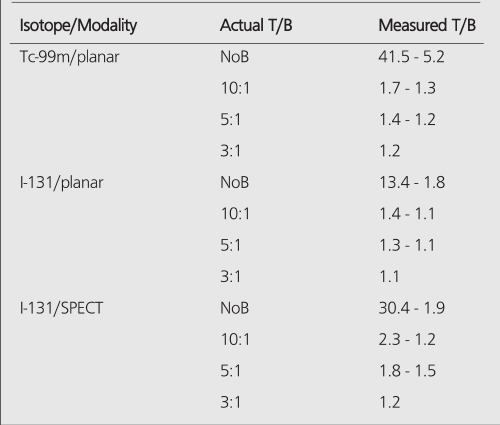
Actual and measured T/B ratios for I-131 and Tc-99m. Thevariation in the measured values is a function of object size, i.e, T/B valuesare higher in larger spheres

**Table 3:**
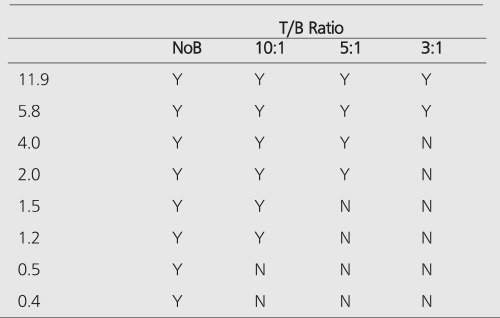
Limits of detection for planar Tc-99m imaging. Based on thesimilar findings in detection for I-131 SPECT and planar modalities,SPECT measurements for Tc-99m were not performed

**Table 4:**
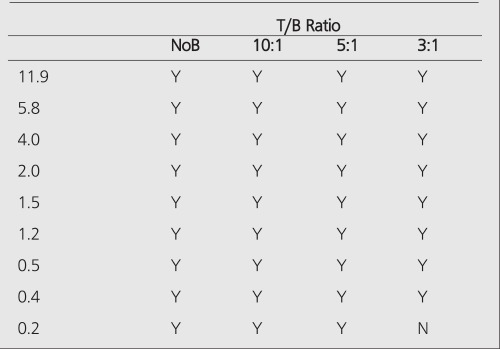
Limits of detectability for a dedicated PET (GE-Advance)scanner (GEMS, Milwaukee, WI) in 2D (septa-in) mode for variousT/B ratios and without background (NoB)

**Table 5:**
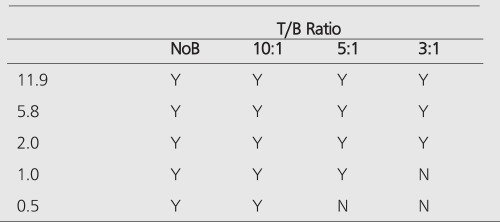
Limits of detection for a gamma camera equipped withMCD (Vertex) (ADAC, Milpitas CA) mode for various T/B ratios andwithout background (NoB)

**Table 6:**
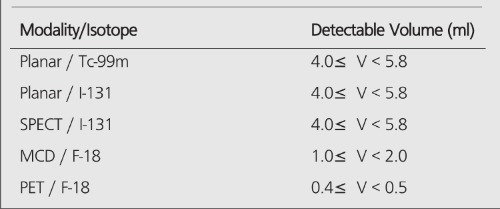
Volume limits of detection with various imaging modalitiesand isotopes. Detectable sphere volume ranges are given for T/Bratio of 3:1
